# An entropy-based framework for genomic variability analysis: A South American case study of human papillomavirus

**DOI:** 10.1371/journal.pone.0349447

**Published:** 2026-05-15

**Authors:** Alan López Leal, Elizabeth Valdés-Muñoz, Jorge Y. Faundez-Acuña, Víctor Rojas-Pérez, Vivían D´ Afonseca, Fabián Silva-Aravena

**Affiliations:** 1 Doctorate in Translational Biotechnology, Faculty of Agricultural and Forestry Sciences, Catholic University of Maule, Talca, Avenida San Miguel, Talca, Chile; 2 Laboratory of Bioinformatics and Computational Chemistry, Department of Translational Medicine, Faculty of Medicine, Catholic University of Maule, Avenida San Miguel, Talca, Chile; 3 Faculty of Medicine, Department of Preclinical Sciences, Parasitology and Microbiology Laboratory, Catholic University of Maule, Avenida San Miguel, Talca, Chile; 4 Faculty of Social and Economic Sciences, Catholic University of Maule, Avenida San Miguel, Talca, Chile; UFRN: Universidade Federal do Rio Grande do Norte, BRAZIL

## Abstract

The genetic diversity of Human Papillomavirus (HPV) poses challenges for molecular detection and genotyping, particularly in regions with distinctive circulating variants such as South America. This study aimed to quantitatively characterize HPV genomic variability using Shannon entropy (H) and to establish a reproducible framework for systematic variability assessment. Positional entropy was calculated across complete and partial HPV genomic sequences and aggregated by gene to compare intra- and intergenotypic conservation patterns using publicly available South American sequences. Intragenotypic analyses showed that most genomic positions were highly conserved (H ≈ 0), consistent with functional constraints. In contrast, intergenotypic comparisons revealed a dual pattern of variability: early genes E5, E6, and E7 displayed higher divergence, whereas capsid genes L1 and L2 were comparatively conserved. These patterns align with known functional and evolutionary constraints across HPV genes. Rather than proposing diagnostic implementations, these results provide a quantitative basis for the preliminary prioritization of conserved and variable genomic regions. Entropy profiling provides a quantitative framework for characterizing genomic variability and may support the identification of conserved and variable regions for future investigation and genomic surveillance efforts, although experimental validation is required to determine diagnostic performance. Overall, this work presents a reproducible and data-driven approach for assessing HPV genomic variability and contributes to the understanding of regional viral diversity in South America.

## 1. Introduction

Papillomaviridae family, characterized by being a double-stranded, non-enveloped DNA virus with a genome of approximately 8 kb. The genome is functionally organized into three main regions: two regions that encode viral proteins (early and late) and a non-coding region that regulates viral replication and transcription. The early region (E) encodes proteins involved in viral DNA replication and transcriptional regulation of the viral life cycle. The late region (L) encodes the structural proteins that make up the virion capsid [1–3].

The viral proteins E1 and E2 are mainly involved in genome replication and regulation, while E4 and E5 contribute to its amplification and cell proliferation. The E6 and E7 genes are particularly important, as they act as oncoproteins [2,3] and, together with E5, drive the viral replication and proliferation cycle during infection. Specifically, E6 and E7 promote entry into the S phase of DNA synthesis and inhibit apoptosis, while E5 promotes cell proliferation through the use of growth factors and evasion of the immune system [4]. The coordinated function of these early proteins contributes to viral persistence [5]. The late genes L1 and L2 encode the structural proteins of the capsid, with L1 being the major protein and L2 the minor protein. Both are expressed in the final stages of the viral cycle and are essential for virion assembly. Finally, the non-coding region (LCR) harbors the viral promoters and enhancers, as well as the DNA replication origin [6,7].

Human papillomavirus (HPV) genotypes are classified according to their oncogenic potential into high-risk (HR-HPV) and low-risk (LR-HPV) types. HR-HPV types are closely associated with the development of malignancies such as cervical, anal, and oropharyngeal cancers. Among them, genotypes 16 and 18 are the most prevalent, along with other high-risk types such as 31, 33, 35, 39, 45, 51, 52, 56, 58, 59, 68, 73, and 82. Low-risk HPV types usually cause benign lesions, mainly genital warts. The most representative low-risk genotypes are 6 and 11, in addition to others such as 40, 42, 43, 44, 48, 54, 61, 70, 72, 81, and 89 [8,9].

HPV genomic diversity exhibits clear phylogeographic structure, with viral lineages showing region-specific distributions worldwide. Previous studies have demonstrated that HPV variants are not uniformly distributed across populations and that lineage composition may differ substantially by geographic region. These patterns highlight the relevance of regional analyses when evaluating viral genomic variability and its potential impact on molecular detection strategies [10,11].

Distinct HPV variant lineages have been documented in Latin American populations, including non-European variants of HPV16 and HPV18 that may differ in persistence and oncogenic potential. Regional studies have also reported heterogeneous genotype distributions across Latin American countries, supporting the importance of understanding local HPV genetic diversity [12]

According to recent data from the Pan American Health Organization (PAHO, 2023), human papillomavirus (HPV) is one of the leading causes of cervical cancer in Latin America and the Caribbean. In this region, the incidence rate is approximately 15.1 cases per 100,000 women, and mortality is estimated at 7.7 per 100,000 [13,14]. This pathology consists of a slowly progressing cellular alteration that originates in the epithelium of the cervix, specifically in the transition zone. Over time, infected cells can multiply and extend into the inner layers of the cervix [15,16]. In the national context, cervical cancer is the second leading cause of death among Chilean women aged 20 to 44. The screening method currently used in the health system is the Papanicolaou test (PAP), which is offered free of charge every three years [17]. Screening is recommended from age 30 onwards (using PAP between ages 25–29 as an alternative). It is estimated that nearly 600 Chilean women die each year from this disease, which is equivalent to two women dying every day, with approximately 1,500 new diagnoses each year. With respect to incidence, Chile ranks seventh worldwide. Globally, approximately 660,000 new cases and 350,000 deaths were reported in 2022 [15,18].

HPV-16 and HPV-18 genotypes are the main oncogenic agents, responsible for most high-grade cervical and anogenital cancers. Their pathogenic activity is based on key mechanisms such as genomic integration and immune evasion. HPV-16, which is also highly prevalent in male urogenital infections, has been associated with the induction of oxidative stress and cellular damage, facilitating viral persistence and tumor progression. HPV-18, although it accounts for a smaller proportion of total cervical cancer than HPV-16, is distinctly associated with the development of adenocarcinomas due to its transcriptional activity. In contrast, HPV6 and HPV11 are classified as low-risk types and are not major contributors to cervical cancer, although they have been sporadically associated with other malignancies such as penile and laryngeal cancers [19,20].

Currently, the most widely used screening methods combine Pap smears with molecular tests, such as Hybrid Capture (HC2), the Cobas® 4800 system, and PCR/qPCR assays specifically targeting genotypes 16 and 18. Although many commercial kits include panels covering between 13 and 21 genotypes [21,22], their validation outside the populations where they were developed may limit their performance in recognizing local strains due to the intrinsic genetic variability of HPV. Although these tests are practical and widely implemented, they have limitations in genotypic specificity, detection of co-infections, and characterization of local variants, in addition, to requiring strict quality controls [23,24]. In contrast, Next Generation Sequencing (NGS) offers sensitivity and specificity greater than 95% and allows for comprehensive genomic analysis of HPV... Although NGS is the gold standard in characterization, its high cost, complexity of implementation, and required infrastructure hinder its routine use in mass screening programs. Therefore, a better understanding of HPV genomic variability is important to support future studies on molecular detection and to interpret how conserved and variable regions are distributed across viral genomes. [25–27].

In this study, we characterized HPV genomic variability using entropy-based metrics to identify patterns of conservation and divergence across genotypes. This approach provides a quantitative framework for describing conserved and variable genomic regions and for guiding their prioritization in future studies.

## 2. Materials and methods

### Adapted entropy-based genomic variability workflow

The methodological framework of this study was adapted from previously published ap proaches on viral genomic variability and Shannon entropy–based analysis, particularly the workflow described by Rojas-Pérez & Villegas (2023) [28]. Following this reference, we integrated steps for genomic data preprocessing, multiple-sequence alignment, entropy-based positional variability quantification, and downstream statistical testing. The pipeline was adapted to the context of HPV genotypes, maintaining the computational logic and analytical rigor of the original protocol while modifying specific steps to accommodate HPV genome structure, available sequencing depth, and study objectives.

### Database accession

A comprehensive bioinformatic and genomic analysis of human papillomavirus (HPV) sequences was conducted. All sequences were retrieved from the NCBI Virus database (https://www.ncbi.nlm.nih.gov/labs/virus/) in August 2025 using Tax ID: 173087. The following filters were applied: (i) complete and partial genomes, (ii) geographic region restricted to South America, (iii) host restricted to *Homo sapiens* (Tax ID: 9606), and (iv) isolation sources reported as nasopharynx, cervix, and skin. The geographic restriction was applied to enable a region-focused variability assessment and to minimize confounding effects from global lineage heterogeneity.

Under these conditions, an initial dataset comprising 21 HPV genotypes was obtained: 11, 16, 18, 30, 31, 33, 35, 52, 56, 58, 59, 66, 67, 68, 73, 81, 82, 87, 115, 156, and 256. Sequences were downloaded in FASTA format. Genotypes represented by only a single available genome (11, 30, 81, and 115) were excluded to ensure robust entropy estimation.

To minimize redundancy, duplicate or highly similar sequences originating from the same isolate or BioProject were identified based on sequence metadata (e.g., accession number, isolate information, and project identifiers) and removed prior to alignment, ensuring a more balanced representation of genomic variability.

After applying these filtering and curation steps, the final dataset comprised 16 genotypes: 16, 18, 31, 33, 35, 52, 56, 58, 59, 66, 67, 68, 73, 82, 87, and 156. The final genotype set was consistently used across all analyses, tables, figures, and supplementary materials. The dataset composition and number of sequences analyzed per genotype are summarized in Table 1.

**Table 1:** Start and end nucleotide positions (genomic coordinates) for early (E1–E7) and late (L1–L2) genes for each HPV genotype analyzed in this study. Gene boundaries were obtained from reference genomes curated in the Papillomavirus Episteme (PaVE) database and were used to map gene regions onto multiple sequence alignments for entropy-based variability analyses. “N.A.” indicates that the gene was not annotated or not present in the corresponding reference genome.

Genotype	E1	E2	E4	E5	E6	E7	L1	L2
16	865-2814	2756-3853	865-3620	3850-4101	104-559	562-858	5639-7156	4237-5658
18	914-2887	2817-3914	912-3684	3936-4157	105-581	590-907	5631-7123	4244-5632
31	862-2751	2693-3811	862-3578	3816-4070	108-557	560-856	5552-7066	4171-5571
33	879-2813	2749-3810	879-3577	3854-4081	109-558	573-866	5594-7093	4210-5613
35	868-2781	2717-3817	868-3319	3814-4065	110-559	562-861	5601-7109	4211-5620
52	864-2807	2743-3849	864-3613	3933-4160	102-548	553-852	5643-7154	4262-5662
56	895-2805	2747-3862	895-3629	N.A	102-569	572-889	5598-7097	4223-5617
58	883-2817	2753-3829	883-3605	3892-4122	110-559	574-870	5643-7139	4244-5662
59	872-2806	2748-3848	872-3615	3908-4129	55-537	542-865	5606-7132	4231-5625
66	895-2789	2729-3838	895-3605	N.A	102-569	572-889	5647-7158	4272-5666
67	875-2785	2721-3827	875-3591	3900-4121	102-551	563-862	5616-7118	4238-5635
68	823-2745	2687-3784	823-3551	3830-4051	1-477	484-816	5488-7005	4098-5507
73	850-2802	2744-3793	850-3560	3813-4037	102-548	550-843	5494-7005	4083-5510
82	876-2804	2746-3825	876-3601	N.A	102-557	565-867	5570-7081	4168-5589
87	819-2771	2713-3840	819-3607	4172-4315	105-551	527-817	5756-7270	4360-5778
156	805-2631	2567-3766	805-3530	N.A	106-528	525-818	5360-6889	3775-5349

The final dataset, including sequence identifiers and accession numbers, is provided in the Supplementary Material to ensure full reproducibility.

### Sequence alignment

Reference genomes corresponding to each analyzed genotype were obtained from the Papillomavirus Episteme (PaVE) platform (https://pave.niaid.nih.gov/search/search). Sequences were filtered by genotype name, and a curated dataset of reference strains was generated in FASTA format for each HPV genotype.

Multiple sequence alignment (MSA) was performed using MAFFT v7.525 in automatic mode (--auto) with default parameters. MAFFT was selected due to its demonstrated accuracy and computational efficiency in aligning viral genomes with heterogeneous levels of sequence conservation, as well as its widespread use in comparative genomic analyses.

Processing was carried out on a high-performance computing server utilizing 64 CPU cores to optimize computational performance. Resulting alignments were stored in FASTA format, organized by genotype, and subsequently used for genetic diversity analyses

### Genetic diversity analysis

Variability across HPV genomes was quantified using the Shannon entropy index as a measure of genetic diversity. Entropy was calculated at each nucleotide position based on genotype-specific multiple sequence alignments, including both complete and partial genome sequences. To ensure robust estimation, entropy values were computed on a per-position basis using only observed nucleotide frequencies, excluding positions affected by gaps or missing data due to incomplete genome coverage.

The analysis was conducted using BioEdit v5.0.9, employing the internal entropy computation tool H(x). For each alignment, Shannon entropy values were estimated at every nucleotide position and exported in tabular format. Comparative analyses were then performed within each genotype to evaluate intragenotypic variability

### Data processing and statistical analysis

Entropy-derived genetic diversity data were processed using the R programming language v4.4.2 (https://www.R-project.org). Before statistical analysis, a custom R script was implemented to remove gaps generated during sequence alignment, in order to refine the dataset and prevent bias in variability estimates. The native subset() function in R was used to retain only positions in the viral genome exhibiting entropy values ≥ 0.5, a threshold defined as acceptable for determining the magnitude of sequence variability as established by Ramette & Buttigieg (Front. Microbiol. 2014; 5:601).

Shannon entropy was calculated according to the formula:


$$\begin{array}{c} H(x)=\overset{k}{\underset{𝕚=\text{1}}{-\Sigma }}pi(x){\text{log}}_{\text{4}}pi(x) \end{array}$$

where H(x) denotes the normalized Shannon entropy, interpreted here as nucleotide variability at genomic position x; pᵢ(x) represents the relative frequency of nucleotide state i at that position; and k corresponds to the number of nucleotide states considered in the alignment. Positions containing gaps or missing data were excluded from the frequency calculation to avoid bias derived from incomplete genome coverage. The logarithm base 4 was used to scale entropy values between 0 and 1.

For each dataset, the following metrics were computed: number of analyzed positions (N_positions), mean entropy (Mean_H), standard deviation (SD_H), quartiles (Q05, Q25, Q75, Q95), minimum entropy (Min_H), maximum entropy (Max_H), and the proportion of genomic positions classified as conserved (% conserved; H = 0), intermediate variability (% medium; 0.1 < H ≤ 0.5), and high variability (% high; H > 0.5).

Additionally, the entropy coefficient of variation (H_cv) was calculated as


$$\begin{array}{c} Hcv=\;\frac{SD\;(H)}{Mean\;(H)} \end{array}$$

Additionally, two complementary indicators were calculated: the **coefficient of variation**


$$\begin{array}{c} Cons_{index}=\text{1}-H \end{array}$$

### Inter-genotype consensus sequence analysis

To compare patterns across genotypes, a multiple sequence alignment (MSA) of consensus genomes was generated using the Biostrings package in R. From the outputs files (tables generated per genotypes), two reciprocal mappings were constructed for each sequence:

(i) MSA column → genome position, and (ii) genome position → MSA column. This bidirectional mapping allowed the projection of gene ranges (start–end) defined for each genotype onto their corresponding homologous columns in the global MSA.

Reference gene boundaries (start–end positions) used for annotation and mapping were obtained from the PaVE database and are summarized in Table 1.

For each aligned column, an inter-genotype cutoff approach was applied: if ≥60% of genotypes with data agreed on the annotated gene, the column was assigned that gene label; otherwise, it was classified as Ambiguous, while columns without data were labeled Unannotated. Ambiguous and unannotated regions were excluded from downstream analyses, as only well-defined gene regions were considered informative. This procedure generated a consensus gene annotation table across the entire MSA. Using the resulting inter-genotype dataset (containing Position, Entropy, Gene, and Group information for the consensus alignment), variability metrics were estimated per gene following the same parameters used for the intra-genotype analysis. These results were visualized through various R-based graphical representations.

### Phylogenetic analysis

To contextualize overall sequence divergence, a maximum likelihood (ML) phylogeny was inferred from the consensus sequences using the ape and phangorn R packages. Sequences were first converted to phyDat format, and an initial tree was constructed via the Neighbor-Joining (NJ) method. Model selection between HKY and GTR, with or without Γ/I components, was performed using the Akaike Information Criterion (AIC/AICc).

The ML tree was then optimized using the optim.pml() function. When appropriate, non-parametric bootstrap analysis was used to evaluate branch support. Genotypes were subsequently clustered using hierarchical clustering (Ward.D2 method) based on the cophenetic distance matrix derived from the ML tree. Clusters were trimmed to a manageable number to enhance visual interpretation. Phylogenetic trees were visualized using ggtree, with clade groupings annotated via groupOTU(), and additional elements such as bars or genotype-specific heatmaps were added using ggtreeExtra.

All scripts and code used for entropy calculation and data processing are publicly available in a GitHub repository maintained by https://github.com/AlanLopez19/HPV-entropy-analysis.

## 3. Results

### High genome-wide conservation revealed by Shannon entropy analysis

**Fig 1:**
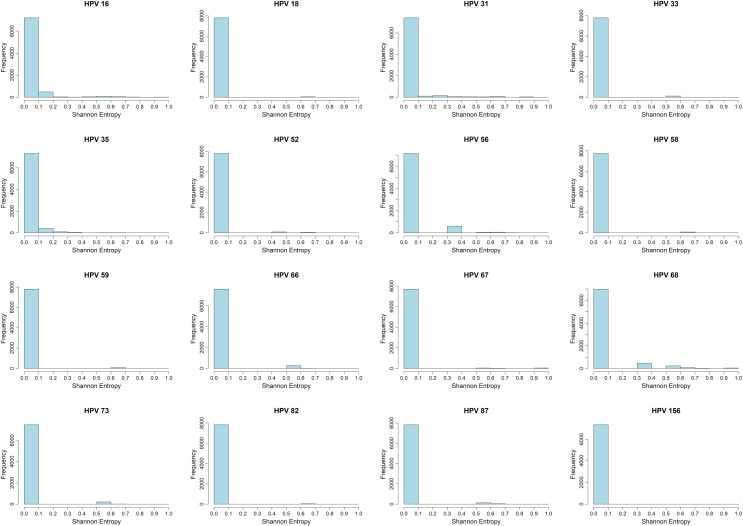
Shannon entropy (H) distributions across genome positions for each HPV genotype. Most sites cluster at low H (high intragenotype conservation), with sparse right-tail bins indicating variable regions. These profiles provide a baseline to identify conserved genomic regions and variable windows that may be informative for genotype differentiation.

The distribution of Shannon entropy (H) was calculated positionally for each HPV genotype based on intra-genotype alignment (Fig 1). The uniform scale of the X-axis (0–1) facilitates direct comparison of variation profiles between genotypes. Predominantly, the distributions are concentrated around values close to zero, with a sharp peak in the first interval (0−0.1). This pattern indicates that most positions, within each genotype, are highly conserved, which is consistent with functional constraints limiting changes in large portions of the genome.

However, subtle differences between genotypes can be observed in the high H value segment. In some genotypes, notable counts are seen in H≈0.2−0.4 and even isolated events with higher values, revealing pockets of local nucleotide diversity. In contrast, in others, the distribution is almost entirely confined to H≈0, suggesting almost total conservation in the cohort analyzed. These inter-genotype variations suggest that the diversity load is not uniform and is predictably concentrated in particular regions or genes. From a methodological and applied point of view, the predominance of H≈0 supports the identification of conserved genomic regions that may warrant further evaluation in future molecular studies. Conversely, higher H values indicate regions that may be informative for genotype differentiation, provided that intra-genotype variation does not compromise consistency. This scenario justifies conducting complementary analyses by gene, inter-genotype comparisons, and inspections with local windows to prioritize specific subregions.

### Intergenotype variability at the gene level based on Shannon entropy

**Fig 2:**
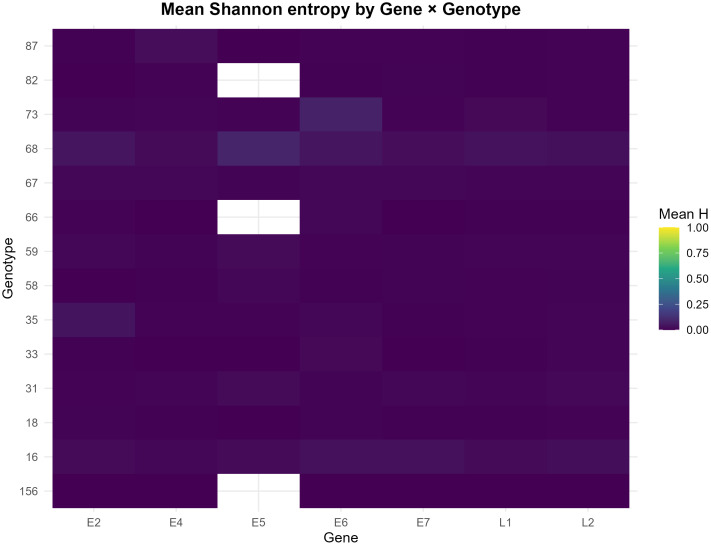
Heatmap of mean Shannon entropy (Mean H; 0–1) by gene (E2, E4, E5, E6, E7, L1, L2) and HPV genotype. Each cell shows the average H computed over all positions annotated for that gene within the genotype. Color scale: low values (purple) = higher conservation; high values (green/yellow) = greater variability. White cells indicate missing data/lack of gene annotation.

Fig 2 summarizes inter-genotype variability at the gene level using Shannon’s mean entropy (Mean H, 0−1), calculated from the alignment of consensus sequences. Specifically, each cell in the matrix represents the average H of all annotated positions for a gene within a given genotype. The color scale indicates conservation (low values, e.g., purple) versus variability (high values, e.g., green/yellow). Blank cells indicate the absence of data or annotation for that gene-genotype pair. Together, this representation synthesizes the genomic heterogeneity between genotypes in a single matrix, respecting the functional structure of the viral genome.

A quantitative summary of gene-level entropy metrics across genotypes is provided in S2 Table.

The figure illustrates the intergenotypic divergence of HPV at the gene level using Shannon’s mean entropy (Mean H, 0−1), calculated from the alignment of consensus sequences. Each cell represents the Mean H of all annotated positions of a specific gene within a genotype; the color scale encodes conservation (low values, e.g., purple) versus variability (high values, e.g., green/yellow). Blank cells denote the absence of data or annotation. On a global scale, a low entropy signal predominates in most gene-genotype pairs, which is consistent with the high intergenotypic conservation of the viral genome. Consistent with the structural biology of the virion, the L1 and L2 genes consistently exhibit lower entropy values relative to early genes. This pattern indicates higher relative conservation at the inter-genotype level and suggests that these regions may warrant further evaluation in future molecular studies.

In contrast, relative increases in H are observed in early genes, mainly E6 and, to a lesser extent, E2/E4, in specific subsets of genotypes. Although the absolute magnitudes remain moderate when averaged by gene, these shifts indicate focused intergenotypic divergence and highlight genomic regions that may be informative for future genotype differentiation studies. The selection of short windows with high intergenotypic H but low intragenotypic variation may offer a useful balance between variability and consistency at the sequence level. Missing cells (e.g., E5 in certain genotypes) reflect annotation or coverage gaps and underscore the need for local inspection and experimental verification prior to diagnostic implementation. In summary, the observed pattern supports an interpretative framework in which conserved regions in L1/L2 and more variable regions in E6/E2/E4 can be comparatively evaluated in future studies of detection and genotype differentiation.

### Quantitative analysis of intragenotypic conservation

**Fig 3:**
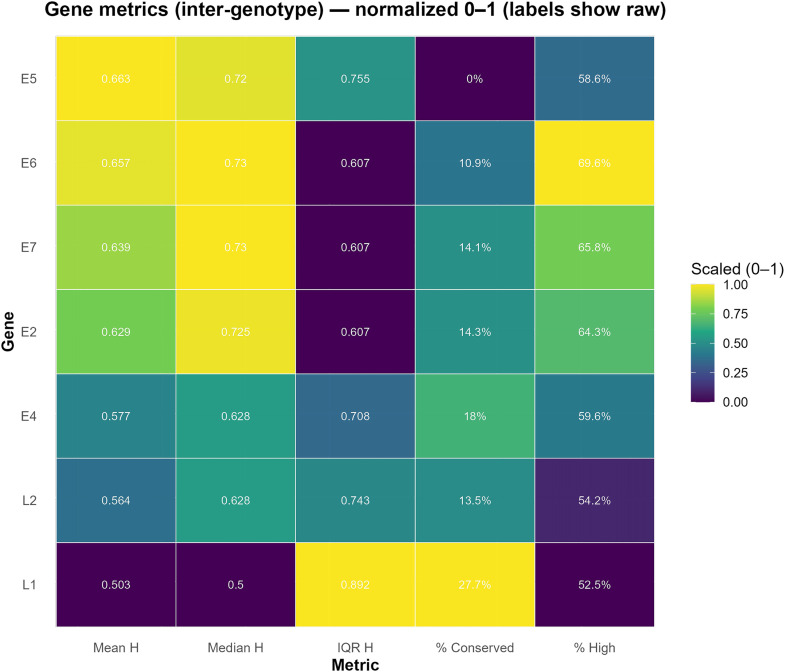
Inter-genotype metrics per gene based on Shannon entropy. Rows = genes (E2, E4, E5, E6, E7, L1, L2); columns = metrics: Mean H (mean entropy H across all annotated positions of the gene across genotypes), Median H (median H), IQR H (Interquartile Range = Q3 − Q1), which quantifies the central dispersion of H and is robust to extreme values; % Conserved (percentage of positions with H = 0) and % High (percentage with H > 0.5). The color scale is normalized by column (0–1), the numerical labels show the raw (unnormalized) values. In this summary, high Mean/Median H values together with high % High and low % Conserved indicate genes with inter-genotype divergence, while low values and high % support these genes as relatively conserved regions across genotypes.

Supplementary S3 Table provides a quantitative summary of intragenotype conservation, detailing the descriptive statistics of Shannon entropy (H) for each genotype and gene. These include the number of positions analyzed (N_positions), the mean, median, standard deviation (SD_H), quantiles (Q05, Q25, Q75, Q95), and minimum (Min_H) and maximum (Max_H) entropy values. In addition, key metrics for characterizing sequence variation are reported, including the percentage of conserved positions (H = 0), positions with intermediate variation (0.1 < H ≤ 0.5), and positions with high variation (H > 0.5), as well as the entropy coefficient of variation (H_cv) and a conservation index scaled from 0 to 1. Together, these metrics enable the characterization of intragenotype conservation and the identification of variability hotspots at the gene level, providing a robust quantitative basis for prioritizing genomic regions for future investigation Fig 3.

The L1 and L2 capsid genes exhibit the lowest mean entropy (Mean H) values and percentages of conserved positions close to 97–100% in most of the genotypes analyzed (HPV 16,18,31,33,35,58,59,66,67,68,73,82,87,156).

This pattern supports their characterization as relatively conserved regions across genotypes; however, the presence of non-zero IQR/MAD in some cases (e.g., L1 in HPV 16) suggests internal heterogeneity and recommends the selection of specifically “flat” sub-windows in the H profile to ensure sustained conservation. In contrast, the E6 and E7 genes showed relative increases in variability, with notable peaks in E6 of HPV 73 (high Mean H and >10% high variation, %_high) and moderate elevations in E6 of HPV 68, which suggests that they may represent informative regions for future typing studies, provided that intra-genotype variation remains low at the window scale. The E5 gene showed markedly heterogeneous behavior: it was close to zero in HPV 18, 33, and 87, but highly variable in HPV 68-E5 (Mean H≈0.09; %_high ≈10%), suggesting a region of potential interest for genotype-specific analyses.

The E2 and E4 genes remained generally conserved, although HPV 35-E2 and HPV 87-E4 showed selective elevations that could be complementary in typing panels. In terms of overall characterization, HPV 156 was the most conserved (Cons_index≈1), while HPV 68 had the highest Mean H in multiple genes (E2, E5, E6, and L1/L2), ranking among the most variable in the cohort. Based on these findings, a combined interpretative framework can be considered, in which conserved regions (L1/L2) and more variable regions (e.g., E6/E7 and E2/E4/E5) are jointly evaluated for their potential relevance in future studies of detection and genotype differentiation.

### Comparison of conservation patterns between early and late genes

**Fig 4:**
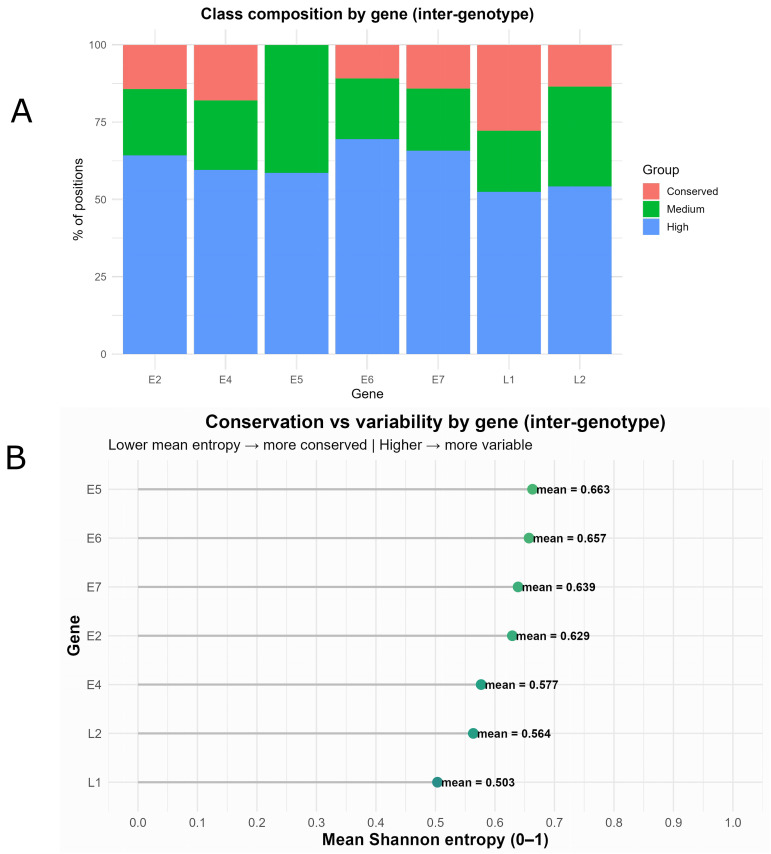
Intergenotype variability per gene in the consensus MSA. **(A)** Percentage of positions by Shannon entropy category (Conserved: H = 0; Intermediate: 0.1–0.5; High: H > 0.5) for each gene (E2, E4, E5, E6, E7, L1, L2). A higher proportion of “High” indicates greater divergence between genotypes. **(B)** Mean entropy (0–1) per gene, with points labeled by their corresponding values. Early genes (E5, E6, and E7) show the highest means and therefore greater variability, whereas capsid genes (L1 and L2) display the lowest mean entropy values, indicating greater relative conservation.

Fig 4 provides a comparative summary of the variability between genotypes at the gene level. In Fig 4A, each stacked bar represents the percentage of positions in a gene classified as Conserved (H=0), Intermediate (0.1<H≤0.5), or High Variation (H>0.5) in the consensus sequence alignment. Overall, the largest fraction of sites is concentrated in the High Variation category, indicating broad inter-genotype divergence across the genome. This pattern is particularly marked in the E5, E6, and E7 genes, where the proportion of positions with H>0.5 clearly exceeds that of conserved or intermediate variation sites. In contrast, L1 and L2 exhibit a relatively smaller fraction of sites with High Variation and somewhat higher percentages of sites with Intermediate or Conserved variation, suggesting greater relative conservation of capsid proteins compared to early genes.

Fig 4B ranks genes according to their mean Shannon entropy (0–1) and displays the corresponding values. The highest entropy values in E5 (~0.66), E6 (~0.66), and E7 (~0.64) indicate that these regions are, on average, the most variable across genotypes, while E2 and E4 occupy intermediate positions. In contrast, L1 (~0.50) and L2 (~0.56) exhibit the lowest mean entropy values among the analyzed regions, supporting their relative conservation compared to early genes, despite exhibiting moderate absolute entropy values.

Taken together, both panels show that early genes (particularly E5, E6, and E7) account for a substantial proportion of inter-genotype variation, whereas capsid regions (L1 and L2) are comparatively more conserved These patterns suggest that L1 and L2 are comparatively conserved regions, whereas early genomic regions may be more informative for future studies of genotype differentiation.

**Fig 5:**
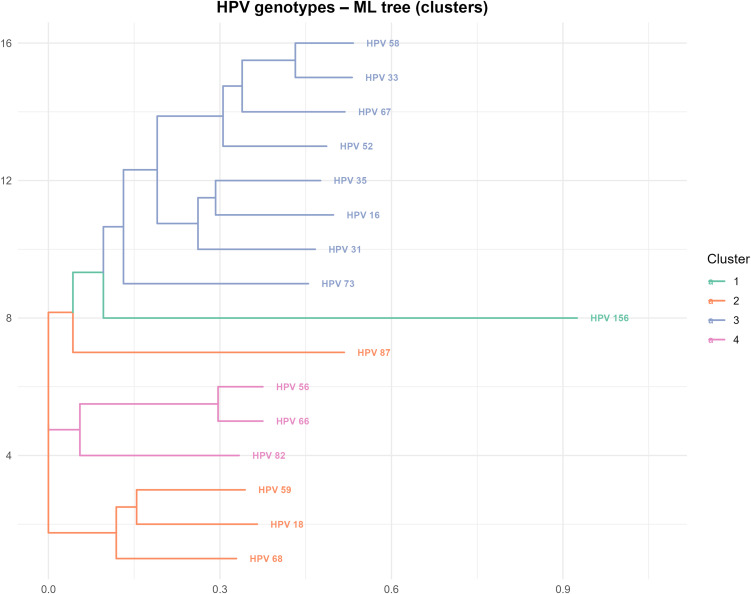
Maximum likelihood tree inferred from the alignment of consensus sequences of the HPV genotypes analyzed, representing their overall sequence divergence. The tree was grouped using hierarchical clustering (Ward.D2) into four main clusters (colors) to contextualize intergenotypic variability. Cluster 1 (Green): HPV 156 genotype, showing the greatest basal divergence. Cluster 3 (Blue): The densest and most recent group, grouping closely related oncogenic genotypes (HPV 16, 31, 33, 35, 52, 58, 67, 73). Clusters 2 (Orange) and 4 (Pink): Intermediate groups containing the remaining oncogenic genotypes. The phylogenetic structure supports the need for highly specific differential markers due to the evolutionary proximity between high-risk genotypes.

The phylogenetic analysis, summarized in Fig 5 using a Maximum Likelihood (ML) tree, illustrates the overall evolutionary relationships among HPV genotypes, which were hierarchically grouped into four main clusters. Cluster 1 emerges as a basal, monogenotypic branch represented solely by HPV 156, whose marked phylogenetic divergence is consistent with its low intragenotypic variability (Cons_index ≈ 1) observed previously.

In contrast, Cluster 3 (Blue) is the densest, grouping high-risk genotypes such as HPV 16,31,33,35,52,58,67, and HPV 73, whose close genetic proximity indicates greater similarity between them, a factor that highlights the importance of identifying genomic regions with sufficient discriminatory variability (such as E6/E7) for their accurate typing. Clusters 2 (Orange: HPV 18,59,82,68) and 4 (Pink: HPV 56,66,87) complete the tree structure, with intermediate evolutionary relationships between each other and with respect to the main group. Taken together, this phylogenetic structure provides an evolutionary framework is consistent with the observed patterns of genomic variability, highlighting that the evolutionary proximity between oncogenic genotypes increases the importance of identifying regions with sufficient intergenotypic variability for future classification-oriented studies.

## 4. Discussion

This study used Shannon entropy (H) as a quantitative metric to evaluate the genomic variability of human papillomavirus (HPV). This bioinformatics approach enables the objective identification of genomic conservation and variability patterns without extending beyond the scope of the observed data [29,30]. To this end, H was calculated positionally along the genome, and the values were aggregated per gene to characterize intra- and intergenotypic conservation. While HPV genomic variability is widely recognized, systematic quantitative comparisons across genes using reproducible entropy-based metrics remain limited. The strength of entropy-based approaches lies in their interpretability and reproducibility rather than methodological complexity.

The histograms obtained (Fig 1) showed that, at the intragenotype level, the distribution of H is predominantly concentrated around H≈0, indicating high genomic conservation consistent with strong functional constraints. However, some genotypes presented discrete right tails (H>0.1), suggesting the presence of localized regions of variability that may warrant further regional analysis [31].

At the intergenotype level (Fig 4), marked differences were observed across genes. The E5, E6, and E7 genes exhibited higher levels of intergenotypic variability (higher Mean H and interquartile ranges), whereas the L1 and L2 capsid genes showed lower entropy values and a higher proportion of conserved positions (H=0). This relative conservation of L1 and L2 is consistent with previous studies reporting comparable diagnostic performance between L1-targeted assays and E6/E7-based assays in different neoplastic contexts [32].

Although L1 is widely used in molecular detection assays, it is important to consider that genomic alterations associated with viral integration events may affect its detection in certain biological contexts [33]. In contrast, the variability observed in E6 and E7 genes is consistent with previous studies describing variants with differential effects on oncogenicity and viral persistence. For example, Antaño-Arias et al. (2021) reported HPV16 E6/E7 variants associated with cervical carcinoma in women from southern Mexico [34], while Zhingre et al. (2023) described differential expression patterns of E6/E7 in HPV18-related cancers that may reflect site-specific viral adaptation [35]. These findings support the biological relevance of variability in early genes beyond simple sequence divergence.

From an evolutionary and epidemiological perspective, these results are consistent with previous studies emphasizing the importance of intragenotypic genomic diversity. Mirabello et al. (2016) demonstrated that HPV16 sublineages are associated with differential cancer risk globally, including in Latin America [36]. Similarly, Burk et al. (2013) highlighted that HPV genomic heterogeneity is unevenly distributed across regions and genes, influencing viral evolution and pathogenesis [37].

Based on the entropy patterns observed, this study provides a reproducible methodological framework for the systematic assessment of genomic variability. To ensure robust characterization, multiple complementary metrics were considered, including mean and median H, interquartile range, median absolute deviation (MAD), percentage of conserved positions (H=0), and a conservation index defined as 1−mean(H). These measures enable a multidimensional description of variability patterns across genomic regions [38].

Regions within L1 and L2 exhibiting low entropy and narrow variability ranges correspond to relatively stable genomic segments, whereas regions within E5, E6, and E7 show higher intergenotypic variability. These patterns illustrate how entropy profiling can be used to describe the distribution of conserved and variable regions across the HPV genome. However, experimental validation is required to determine the functional or diagnostic relevance of these observations. This study focused on genome-wide variability patterns rather than individual polymorphism characterization, which remains an important area for future research.

The broader relevance of these findings lies in the interpretation of genomic variability patterns. The observed distribution of conserved and variable regions may contribute to a better understanding of HPV genomic diversity in South America and its potential implications for molecular detection approaches. Given that many commercial assays have been developed using non-Latin American reference sequences, regional genomic variability may influence their performance in specific populations [39–40].

This study has several limitations that should be considered. First, the analysis relies exclusively on publicly available genomic sequences, which may introduce sampling biases, uneven genotype representation, and potential residual redundancy despite identity-based filtering and metadata curation. Second, the inclusion of both complete and partial genomes, although controlled through coverage-based filtering, may still affect variability estimates in genomic regions with lower sequence representation. Finally, the findings are based on computational analyses and descriptive metrics; therefore, experimental validation is required to assess the functional and diagnostic relevance of the identified regions.

## 5. Conclusions

This study highlights the utility of Shannon entropy as a quantitative and reproducible approach for characterizing HPV genomic variability based on South American sequences. The results reveal a dual pattern of variability, with high intragenotypic conservation and greater intergenotypic divergence, particularly in early genes such as E5, E6, and E7, whereas capsid genes L1 and L2 remain comparatively more conserved.

These findings provide a systematic framework for interpreting conserved and variable genomic regions that may be relevant for future studies on molecular detection and genomic surveillance. Regions with low entropy correspond to stable genomic segments, whereas more variable regions may be informative for genotype differentiation. However, experimental validation is required to confirm their potential applicability in diagnostic contexts.

Overall, this work establishes a reproducible, data-driven framework for HPV genomic variability assessment that can support future research on molecular detection strategies and contribute to a better understanding of regional viral diversity.

## Supporting information

S1 Table Composition of the HPV genomic dataset analyzed in this study. List of all HPV sequences retrieved from the NCBI Virus database, including genotype, accession number, country or region of origin, isolation source, and genome completeness. This dataset served as the basis for all entropy analyses.
(XLSX)

S2 Table Intergenotypic entropy metrics across HPV genes. Gene-level entropy summaries derived from consensus alignments across genotypes, including mean entropy, median entropy, interquartile range, median absolute deviation, and percentages of conserved and highly variable positions.
(XLSX)

S3 Table Shannon entropy–based metrics of intragenotype sequence conservation by genotype and gene. Descriptive statistics of positional Shannon entropy (H) within each genotype, including number of analyzed positions, mean, median, standard deviation, quantiles, minimum and maximum values, percentage of conserved positions (H = 0), intermediate variability (0.1 < H ≤ 0.5), high variability (H > 0.5), entropy coefficient of variation, and conservation index.
(XLSX)

## Abbreviations

HPV: Human Papillomavirus
qPCR: Quantitative Polymerase Chain Reaction
PCR: Polymerase Chain Reaction
NGS: Next-Generation Sequencing
MSA: Multiple Sequence Alignment
ML: Maximum Likelihood
AIC/ AICc: Akaike Information Criterion/ Akaike Information Criterion corrected
IQR: Interquartile Range
LCR: Long Control Region
HPC: High-Performance Computing
PaVE: Papillomavirus Episteme
NCBI: National Center for Biotechnology Information
H(x): Shannon entropy at position x
